# Maternal pre-pregnancy BMI and reproductive health in adult sons: a study in the Danish National Birth Cohort

**DOI:** 10.1093/humrep/dead230

**Published:** 2023-11-04

**Authors:** Anne Gaml-Sørensen, Anne Hjorth Thomsen, Sandra Søgaard Tøttenborg, Nis Brix, Karin Sørig Hougaard, Gunnar Toft, Siri Eldevik Håberg, Mikko Myrskylä, Jens Peter Bonde, Cecilia Høst Ramlau-Hansen

**Affiliations:** Department of Public Health, Research Unit for Epidemiology, Aarhus University, Aarhus, Denmark; Department of Public Health, Research Unit for Epidemiology, Aarhus University, Aarhus, Denmark; Department of Occupational and Environmental Medicine, Copenhagen University Hospital—Bispebjerg and Frederiksberg, Copenhagen, Denmark; Department of Public Health, Faculty of Health Sciences, University of Copenhagen, Copenhagen, Denmark; Department of Public Health, Research Unit for Epidemiology, Aarhus University, Aarhus, Denmark; Department of Clinical Genetics, Aarhus University Hospital, Aarhus, Denmark; Department of Public Health, Faculty of Health Sciences, University of Copenhagen, Copenhagen, Denmark; National Research Centre for Working Environment, Copenhagen, Denmark; Steno Diabetes Center Aarhus, Aarhus University Hospital, Aarhus, Denmark; Centre for Fertility and Health, Norwegian Institute of Public Health, Oslo, Norway; Max Planck Institute for Demographic Research, Rostock, Germany; Center for Social Data Science and Population Research Unit, University of Helsinki, Helsinki, Finland; Max Planck—University of Helsinki Center for Social Inequalities in Population Health, Rostock, Germany; Department of Occupational and Environmental Medicine, Copenhagen University Hospital—Bispebjerg and Frederiksberg, Copenhagen, Denmark; Department of Public Health, Faculty of Health Sciences, University of Copenhagen, Copenhagen, Denmark; Department of Public Health, Research Unit for Epidemiology, Aarhus University, Aarhus, Denmark

**Keywords:** semen quality, testes volume, reproductive hormones, foetal programming, oestrogen hypothesis, maternal overweight, maternal obesity, mediation analysis

## Abstract

**STUDY QUESTION:**

Is maternal pre-pregnancy BMI associated with semen quality, testes volume, and reproductive hormone levels in sons?

**SUMMARY ANSWER:**

Maternal pre-pregnancy BMI was associated with an altered reproductive hormone profile in young adult sons, characterized by higher levels of oestradiol, LH, and free androgen index (FAI) and lower levels of sex hormone-binding globulin (SHBG) in sons born of mothers with pre-pregnancy overweight and obesity.

**WHAT IS KNOWN ALREADY:**

Evidence suggests that maternal pre-pregnancy BMI may influence reproductive health later in life. Only one pilot study has investigated the association between maternal pre-pregnancy BMI and reproductive health outcomes in sons, suggesting that a high BMI was associated with impaired reproductive function in the adult sons.

**STUDY DESIGN, SIZE, DURATION:**

A population-based follow-up study of 1058 young men from the Fetal Programming of Semen Quality (FEPOS) cohort nested within the Danish National Birth Cohort (DNBC), 1998–2019, was carried out.

**PARTICIPANTS/MATERIALS, SETTING, METHODS:**

In total, 1058 adult sons (median age 19 years, 2 months), born 1998–2000 by mothers included in the DNBC, participated in FEPOS. At a clinical examination, they provided a semen and blood sample, measured their testes volume, and had height and weight measured. Maternal pre-pregnancy BMI was obtained by self-report in early pregnancy. Semen characteristics, testes volume, and reproductive hormone levels were analysed according to maternal pre-pregnancy BMI categories and as restricted cubic splines using negative binomial and ordinary least square regression models. Mediation analyses examined potential mediation by the sons’ birthweight, pubertal timing, fat mass, and BMI. Additional analyses investigated the role of paternal BMI in the potential associations between maternal BMI and reproductive health outcomes.

**MAIN RESULTS AND THE ROLE OF CHANCE:**

We found no consistent associations between maternal pre-pregnancy BMI and semen characteristics or testes volume. Sons of mothers with higher pre-pregnancy BMI had higher oestradiol and lower SHBG levels, both in a dose-dependent manner. Sons of mothers with pre-pregnancy obesity (≥30 kg/m^2^) had higher LH levels and a higher FAI than sons born by mothers with normal pre-pregnancy BMI (18.5–24.9 kg/m^2^). The mediation analyses suggested that the effect of maternal pre-pregnancy BMI on higher levels of oestrogen, LH, and FAI was partly mediated by the sons’ birthweight, in addition to adult fat mass and BMI measured at the clinical examination, whereas most of the effect on lower levels of SHBG was primarily mediated by the sons’ own fat mass and BMI. Paternal BMI was not a strong confounder of the associations in this study.

**LIMITATIONS, REASONS FOR CAUTION:**

This study was based in a population-based cohort with a low prevalence of overweight and obesity in both mothers and adult sons. Some men (10%) had blood for reproductive hormone assessment drawn in the evening. While several potential confounding factors were accounted for, this study's inherent risk of residual and unmeasured confounding precludes provision of causal estimates. Therefore, caution should be given when interpreting the causal effect of maternal BMI on sons’ reproductive health.

**WIDER IMPLICATIONS OF THE FINDINGS:**

Given the widespread occurrence of overweight and obesity among pregnant women, it is imperative to thoroughly examine the potential consequences for reproductive hormone levels in adult sons. The potential effects of maternal pre-pregnancy obesity on sons’ reproductive hormone profile may potentially be partly avoided by the prevention of overweight and obesity in the sons.

**STUDY FUNDING/COMPETING INTEREST(S):**

The project was funded by the Lundbeck Foundation (R170-2014-855), the Capital Region of Denmark, Medical doctor Sofus Carl Emil Friis and spouse Olga Doris Friis's Grant, Axel Muusfeldt's Foundation (2016-491), AP Møller Foundation (16-37), the Health Foundation, Dagmar Marshall's Fond, Aarhus University, Independent Research Fund Denmark (9039-00128B), and the European Union (ERC, BIOSFER, 101071773). Views and opinions expressed are, however, those of the authors only and do not necessarily reflect those of the European Union or the European Research Council. Neither the European Union nor the granting authority can be held responsible. The authors declare that they have no conflict of interest.

**TRIAL REGISTRATION NUMBER:**

N/A.

## Introduction

Around 15–20% of couples of reproductive age are affected by infertility (Center for Disease Control and Prevention, 2022; World Health Organization, 2022b), and up to half of these cases may be explained by male factor infertility (Sharma *et al.*, 2021). Consequently, the use of medically assisted reproduction is increasing in most Western countries (Ferraretti *et al.*, 2017; Dansk Fertilitetsselskab, 2021). Notably, the inability to father children is strongly related to psychosocial morbidities for the affected individual and to economic challenges for society, making impaired male reproductive health a major public health concern (Trasande *et al.*, 2015; Sylvest *et al.*, 2018; Levine *et al.*, 2023).

The causes of impaired male reproductive health remain largely unknown (Sharma *et al.*, 2021). However, exposures in foetal life may be of particular importance for later reproductive health (Sharpe and Skakkebaek, 1993; Skakkebaek *et al.*, 2016). Building on the Developmental Origins of Health and Disease hypothesis first described by Barker and Osmond (1986), Sharpe and Skakkebaek (1993) proposed that exposure to increased oestrogen levels in foetal life may alter the development of the male reproductive organs and impair later reproductive health. Elevated oestrogen levels initiate negative feedback, leading to a decrease in GnRH release. This reduction in GnRH further lowers LH and FSH secretion, which in turn negatively affects testosterone production (Dwyer and Quinton, 2019). The hypothesis suggests that exposure to higher foetal oestrogen levels might perpetuate these disruptions, potentially influencing reproductive health in adulthood (Sharpe and Skakkebaek, 1993). The proposed oestrogen hypothesis resulted in a number of studies on male reproductive health using various proxies of elevated maternal oestrogen levels, such as twin pregnancies (Storgaard *et al.*, 2002) and maternal age at menarche as a proxy for elevated oestrogen levels during pregnancy (Langergaard *et al.*, 2023).

Maternal pre-pregnancy overweight and obesity may also be used as a proxy for elevated oestrogen levels during pregnancy (Ramlau-Hansen *et al.*, 2007). Obesity and overweight are associated with elevated adipose tissue resulting in an augmented production of oestradiol (Pasquali, 2006). This, in turn, potentially contributes to an increase in foetal exposure to oestrogen in offspring born of overweight and obese mothers. This highlights the interplay between maternal BMI, oestrogen, and male reproductive health. The prevalence of overweight and obesity among pregnant women is increasing worldwide (Chen *et al.*, 2018), highlighting the importance of investigating potential consequences hereof. This pilot study of 328 young Danish men found indications of lower sperm concentration in sons born to overweight mothers (Ramlau-Hansen *et al.*, 2007). Though the study indicated an association between higher maternal BMI and lower semen quality in sons, the study had low a relatively low sample size and did not distinguish between overweight and obesity in the mothers. Therefore, we aimed to investigate whether maternal pre-pregnancy BMI was associated with reproductive health, assessed as semen characteristics, testes volume, and reproductive hormone levels, in a large population-based cohort of young Danish men from the general population.

## Materials and methods

This study is based on data from the Fetal Programming of Semen Quality (FEPOS) cohort: a male-offspring cohort within the Danish National Birth Cohort (DNBC).

### Study population

From 1996 to 2000, Danish-speaking pregnant women were invited to the DNBC at the first antenatal visit at their general practitioner. More than 90 000 women were recruited, corresponding to 30% of all pregnant women in Denmark during the inclusion period (Olsen *et al.*, 2001). Among several data collections during pregnancy and after birth, the women provided comprehensive information on health and health behaviour in computer-assisted telephone interviews, among others twice during pregnancy and twice after birth, and gave gestational blood samples for storage in the Danish Biobank.

**Figure 1.**
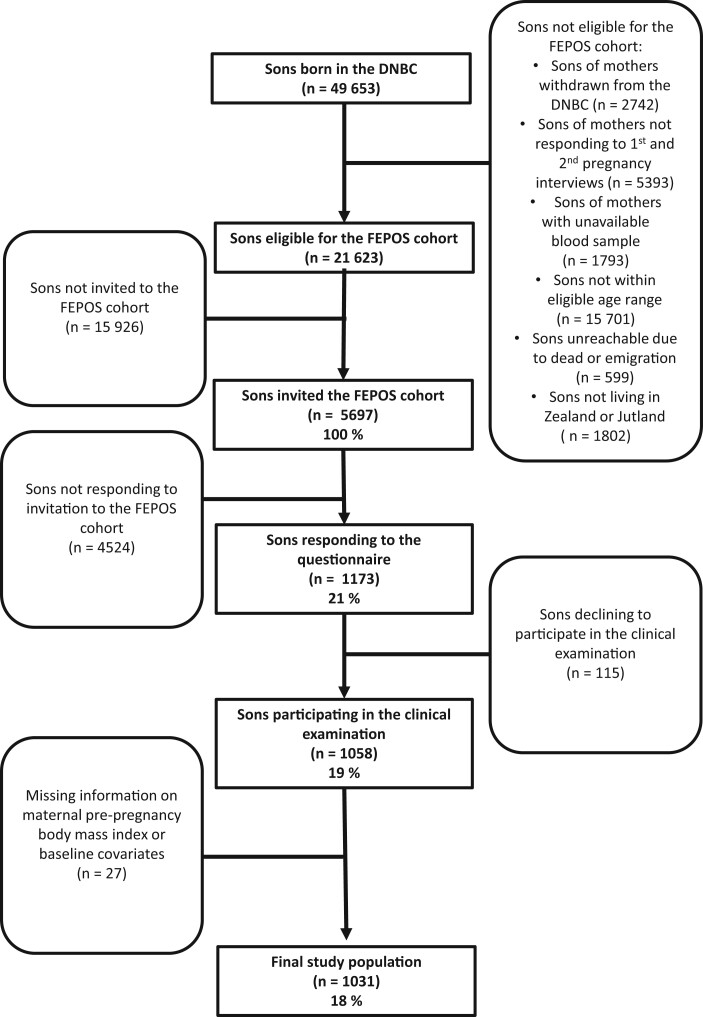
**Flowchart of the inclusion of participants in the study, the Fetal Programming of Semen Quality Cohort within the Danish National Birth Cohort, Denmark, 1998–2019.** Eligible sons were born of mothers from the DNBC who completed two computer-assisted telephone interviews in pregnancy and had a stored gestational blood sample in the DNBC biobank. The sons themselves had to be at least 18 years and 9 months old and live in Zealand or Jutland in proximity to the FEPOS clinics. In total, 5697 were randomly and consecutively invited to participate, 1058 sons (19%) participated in a clinical examination, and 1031 sons (18%) were included in the study: Maternal pre-pregnancy BMI and reproductive health in adult sons: a study in the Danish National Birth Cohort. FEPOS, Fetal Programming of Semen Quality; DNBC, Danish National Birth Cohort.

A total of 49 653 sons were born of mothers from the DNBC. To be considered for participation in FEPOS, sons needed to remain enrolled in the DNBC, have mothers who completed the two computer-assisted telephone interviews, and have a stored gestational blood sample in the DNBC biobank (n = 39 725). Additionally, FEPOS participants had to be at least 18 years and 9 months old during the study period from 2017 to 2019 (n = 24 024). Those who had not passed away or emigrated (n = 23 425), and those living in Zealand (n = 8817) or Jutland (n = 12 806) within relative proximity to the FEPOS clinics, were eligible. This made a total of 21 623 young men eligible for the invitation to participate (Keglberg Hærvig *et al.*, 2020). Of 21 623 eligible sons, 5697 (100%) were randomly and consecutively invited to answer a survey on health and health behaviour. Sons were encouraged to decline participation if they had only one or no testis in the scrotum or had undergone sterilization, orchidectomy, or chemotherapy. In total, 1058 sons (19%) participated in a clinical examination where they gave a semen and a blood sample, measured their testes volume, and had height and weight measured. Among these, 1031 sons (18%) had mothers with information on maternal BMI and baseline covariates, hereby constituting our final study population (Fig. 1).

### Exposure

Pre-pregnancy BMI was calculated from self-reported information on height ('How tall are you?') and pre-pregnancy weight ('What was your weight before the pregnancy?') obtained in the first DNBC interview around gestational week 16. The BMI was calculated as weight (kg)/height (m^2^). Pre-pregnancy BMI was categorized according to the definitions by the World Health Organization (WHO) as underweight (<18.5 kg/m^2^), normal weight (18.5–24.9 kg/m^2^; reference), overweight (25–29.9 kg/m^2^), and obese (≥30 kg/m^2^) (World Health Organization, 2022a).

### Outcomes

Semen characteristics included semen volume, sperm concentration, total sperm count, sperm motility, sperm morphology, DNA fragmentation index (DFI), and high DNA stainability. All measures were performed according to recommendations from WHO 2010 (World Health Organization, 2010), were continuously quality controlled (Keglberg Hærvig *et al.*, 2020), and compared to the Björndahl guidelines (Björndahl *et al.*, 2016). The semen samples were collected at home or in the clinic. When collected at home, the participants were instructed to keep the sample close to the body to ensure a temperature of 37°C during transportation to the clinic. The sons participating in the study were encouraged to abstain from sexual activity for a minimum of 2 days prior to providing their sample. The actual duration of sexual abstinence was recorded during the clinical examination.

At the examination, the sons measured the volume of both testes using a Prader Orchidometer; a method previously validated (Ramlau-Hansen *et al.*, 2007). The average of the two testes was used as the outcome.

Moreover, the sons had non-fasting venous blood samples drawn. Reproductive hormones measured included testosterone, oestradiol, sex hormone-binding globulin (SHBG), FSH, LH, and free androgen index (FAI) calculated as testosterone/SHBG × 100%. Testosterone and oestradiol levels were analysed using liquid chromatography–tandem mass spectrometry (LC–MS/MS). For testosterone, the coefficient of variation (CV) was 7% at 14.1 nmol/l, and the limit of detection (LOD) was 0.12 nmol/l. For oestradiol, the CV was 7.5% at 106 pmol/l and LOD was 15 pmol/l. SHBG, FSH, and LH were measured using immunoassays (Cobas^®^ 8000 e602; Roche Diagnostics, Mannheim, Germany). For SHBG, the CV was 1.1 –1.7% and LOD was 0.350 nmol/l. For FSH and LH, CVs were 2.5–2.8% and 0.7–1.2% respectively, and LOD was 0.1 IU/l. Samples below LOD (87 for oestradiol and <5 for LH and FSH) were replaced by LOD/√2. All outcome assessments are described in detail elsewhere (Gaml-Sørensen *et al.*, 2023) and in Supplementary File S1.

### Covariates

Potential confounding factors were identified using previous literature and directed acyclic graphs (Supplementary Fig. S1) and were included as covariates. Highest parental socioeconomic status (defined according to occupation and level of education derived from the Danish International Standard Class of Occupation and Education codes (ISCO-88 and ISCED)), information on maternal smoking, and alcohol intake in the first trimester was obtained from the first interview in the DNBC. Maternal age at delivery was obtained from the Danish Medical Birth Registry.

Precision variables, i.e. variables expected to be strongly associated with the outcomes under study, were identified in the existing literature and included to increase the precision of the estimates. We included the following precision variables in models of semen characteristics: abstinence time, spillage (participants reporting spillage were excluded from models of volume and total sperm count), place of semen sample collection, and interval from ejaculation to the analysis of the semen sample (only included in models of motility). In models of analysis of testes volume, abstinence time was included, and in models of reproductive hormone levels, time of the day of blood sampling was included. All precision variable information was obtained during the clinical examination.

For mediation analyses, we used information on birthweight, which was obtained from the Danish Medical Birth Register, pubertal timing, which was obtained from the FEPOS survey completed by the sons prior to attending the clinical examination, in addition to the sons’ fat mass and BMI, which were obtained at the clinical examination. Pubertal timing was derived based on the sons’ answer to questions on timing of axillary hair growth, pubic hair growth, facial hair growth, or voice break earlier, at the same time or later than peers. A composite measure was derived, as described previously (Langergaard *et al.*, 2023), where a low score indicated earlier pubertal timing and a high score indicated later pubertal timing. At the clinical examination, the sons had their height (in centimetres) and weight (in kilos) measured using a seca^®^ 213 Height Measure (seca^®^, Hamburg, Germany) and an MC-780MA Body Composition Analyzer (Tanita^®^, Tokyo, Japan), respectively (Keglberg Hærvig *et al.*, 2020). Fat mass was estimated directly by the body scanner and BMI was calculated as weight (kg)/height (m^2^).

For additional analyses, we used information on paternal BMI, which was obtained from a follow-up interview of the mothers 18 months after birth. Some mothers (n = 769) provided information on height and weight of the biological father. Using this information, paternal BMI was calculated as weight (kg)/height (m^2^) and categorized according to the definitions by WHO as underweight (<18.5 kg/m^2^), normal weight (18.5–24.9 kg/m^2^; reference), overweight (25–29.9 kg/m^2^), and obese (≥ 30 kg/m^2^) (World Health Organization, 2022a). However, less than five fathers were underweight, and they were analysed in the normal weight category. All variables were categorized or kept continuous as shown in Table 1.

**Table 1.** Baseline characteristics, potential mediators and precision variables according to categorizations of maternal pre-pregnancy BMI in 1031 participants from the Fetal Programming of Semen Quality Cohort, Denmark, 1998–2019.

Categorical maternal pre-pregnancy BMI (pseudo range, kg/m^2^)	Underweight (16.3–18.5)	Normal weight (18.5–25.0)	Overweight (25.0–29.7)	Obese (30.1–39.4)	Missings %
**N (%)**	64 (6.2)	756 (73.3)	164 (15.9)	47 (4.6)	
**Baseline characteristics**					
**Highest social class of parents**					0
High-grade professional	24 (37.5)	267 (35.3)	51 (31.1)	6 (12.8)	
Low-grade professional	20 (31.3)	252 (33.3)	>54 (>32.9)	15 (31.9)	
Skilled or unskilled worker	15 (23.4)	197 (26.1)	54 (32.9)	26 (55.3)	
Student or economically inactive	5 (7.8)	40 (5.3)	<5 (<3.0)	0 (0.0)	
**Daily number of cigarettes in 1st trimester**					0
Non-smoker	45 (70.3)	586 (77.5)	126 (76.8)	>36 (>76.6)	
0–10 cigarettes/day	14 (21.9)	149 (19.7)	32 (19.5)	6 (12.8)	
>10 cigarettes/day	5 (7.8)	21 (2.8)	6 (3.7)	<5 (<10.6)	
**Alcohol intake in 1st trimester**					0
No	35 (54.7)	389 (51.5)	95 (57.9)	22 (46.8)	
Yes	29 (45.3)	367 (48.5)	69 (42.1)	25 (53.2)	
**Maternal age at delivery (years), mean (SD)**	30.7 (4.2)	31.2 (4.1)	30.5 (4.2)	30.0 (3.7)	0
**Paternal BMI (kg/m^2^)**					25.4
Normal	31 (48.4)	343 (45.4)	70 (42.7)	>16 (>34.0)	
Overweight	10 (15.6)	199 (26.3)	49 (29.9)	14 (29.8)	
Obese	0 (0.0)	23 (3.0)	9 (5.5)	<5 (<10.6)	
**Potential mediators**					
**Birthweight (g), mean (SD)**	3506 (489)	3703 (539)	3803 (533)	3784 (566)	1.5
**Composite marker of pubertal timing, mean (SD)**	2.1 (0.5)	2.1 (0.5)	2.0 (0.5)	2.0 (0.5)	2.2
**Fat mass (kg), mean (SD)**	8.5 (4.8)	11.1 (6.2)	13.2 (7.6)	15.9 (7.9)	<1
**Sons’ own BMI (kg/m^2^)**					<1
Underweight	13 (20.6)	55 (7.3)	8 (4.9)	0 (0.0)	
Normal	>40 (>62.5)	587 (77.7)	107 (65.6)	>25 (>53.2)	
Overweight	6 (9.5)	101 (13.4)	41 (25.2)	17 (36.2)	
Obese	<5 (<7.8)	12 (1.6)	7 (4.3)	<5(10.6)	
**Precision variables**					
**Abstinence time in days**					<1
<2	<32 (<50.0)	250 (33.2)	63 (38.4)	13 (27.7)	
2–3	14 (22.2)	<257 (<34.0)	54 (32.9)	12 (25.5)	
>3	18 (28.6)	249 (33.1)	47 (28.7)	22 (46.8)	
**Spillage**					<1
No	<46 (<71.9)	632 (84.3)	<135 (<82.3)	37 (78.7)	
Yes	18 (28.6)	118 (15.7)	29 (17.9)	10 (21.3)	
**Place of semen sample**					<1
At home	< 5 (<7.8)	102 (13.6)	25 (15.4)	5 (10.9)	
At clinic	> 59 (>92.2)	648 (86.4)	<139 (<87.8)	<42 (<89.4)	
**Interval between ejaculation and time of analysis**					1
≤60 min	<51 (<79.7)	<563 (<74.5)	120 (75.9)	<38 (<80.9)	
>60 min	13 (20.6)	193 (25.7)	38 (24.1)	9 (19.6)	
**Time of the day of blood sampling**					1
Morning	23 (35.9)	277 (37.0)	50 (31.1)	21 (45.7)	
Afternoon	>36 (>56.3)	388 (51.8)	<98 (<59.8)	21 (45.7)	
Evening	<5 (<7.8)	84 (11.2)	16 (9.9)	<5 (<10.6)	

Local data regulations do not allow reporting of numbers below five. Therefore, some numbers in the table have been masked (</>) to hide numbers smaller than 5.

### Statistical analysis

The study population was described according to maternal BMI categorizations using proportions (%) and mean (SD), and the distribution of the reproductive health outcomes according to maternal BMI categorizations was presented as pseudo median with pseudo interquartile range (IQR). The distribution of the reproductive hormone levels according to the time of blood sampling was presented as pseudo median with pseudo IQR.

The data on semen characteristics were non-normally distributed and over-dispersed and were therefore analysed using negative binomial regression models fitted by maximum likelihood estimation (Stata’s *nbreg* command). We estimated crude and adjusted mean ratios with 95% CI for semen characteristics and testes volume according to pre-pregnancy BMI categorizations with normal weight as the reference group. Relative percentage differences were calculated as (ratio − 1) × 100%. The data on reproductive hormone levels were analysed using ordinary linear regression models on ln-transformed data. Estimates were back-transformed and presented as percentage differences with 95% CI relative to normal weight.

We also modelled pre-pregnancy BMI (continuous) as restricted cubic splines with three knots (at 10th percentile: 19.0 kg/m^2^, 50th percentile: 22.1 kg/m^2^, 90th percentile 27.3 kg/m^2^) to inspect potential non-linearity and to visualize associations between maternal pre-pregnancy BMI and the outcomes.

All models were fitted with potential confounding factors and precision variables. Maternal age at delivery was the only continuous variable and was modelled as a second-order polynomial to allow for potential non-linearity. Though the proportion of progressively motile spermatozoa was of primary interest, motility was analysed as the proportion of non-progressive + immotile spermatozoa to ensure optimal model fit. This strategy was chosen since no standard transformation of the proportion of motile spermatozoa yielded an acceptable fit using linear regression, negative binomial regression, or other regression models.

All models were also fitted with inverse probability of selection weights to account for potential differential non-participation (Hernan *et al.*, 2004). We estimated the probability of participation using a logistic regression model with participation (yes, no) in FEPOS and complete information on maternal BMI and baseline covariates as the dependent variable. Selected potential confounding factors (maternal smoking in the first trimester, maternal alcohol in the first trimester, maternal age, and maternal socioeconomic status) and region of participation were chosen *a priori* and used as explanatory factors for participation. The inverse of the individual predicted probability of participation was then assigned to each participant in all analyses to make a pseudo-population representative of all invited to FEPOS. Owing to this re-weighted approach, all models were fitted with robust standard errors.

We checked the semen characteristics models by comparing the observed distributions of the outcomes against the model-based distributions with Q–Q plots and by fitting standardized deviance residuals against the model prediction in rank. Ordinary linear regression models were checked by plotting the model-based residuals in Q–Q plots and by plotting the model-based prediction against expected values. All model checks were satisfactory. All percentiles were calculated and presented as pseudo percentiles (calculated as the mean of the five percentiles closest to the target percentile) to comply with local regulations (General Data Protection Regulation (GDPR), Regulation (EU), 2016/679 of 25 May 2018). Data management and statistical analyses were conducted in STATA 17.0 (StataCorp, College Station, TX, USA).

### Sensitivity analyses

We made sensitivity analyses to assess the robustness of our results towards limitations of the data collection. First, we made a sensitivity analysis of the semen characteristics excluding participants who collected the semen sample at home to consider that their semen samples were transported for up to 1 h before analysis. Second, we made a sensitivity analysis of the reproductive hormone levels excluding participants that had blood drawn in the evening to consider that reproductive hormone levels fluctuate in a circadian rhythm (Brambilla *et al.*, 2009). Third, we made a sensitivity analysis of the semen characteristics not adjusting for abstinence time as a precision variable, since abstinence time could act as a potential mediator in our study, since sons with increased BMI may have impaired sexual functioning (Kolotkin *et al.*, 2012). Lastly, we reran the main analysis without fitting the models with inverse probability of selection weights to assess the influence of these on the results.

### Mediation analyses

Mediation analyses were conducted to explore potential mediation by birthweight, pubertal timing, the son's fat mass, and own BMI. Based on the counterfactual framework to causal mediation analysis, we decomposed the total effect of maternal pre-pregnancy BMI on the outcomes into the direct effect and the indirect effect. The direct effect represents the non-mediated effect, which is the effect of maternal pre-pregnancy BMI on the reproductive health outcomes not mediated by the potential mediator under investigation; hence, it represents the direct association between maternal pre-pregnancy BMI and the outcomes through all other paths than the mediator under investigation. The indirect effect represents the mediated effect, which is the effect of maternal pre-pregnancy BMI on the reproductive health outcomes mediated by the potential mediator under investigation; hence, it represents the indirect association between maternal pre-pregnancy BMI and the outcomes through the mediator under investigation only. We used a regression-based approach (Stata’s *paramed* package). The 95% CI was derived using standard errors obtained by the delta method.

### Additional analyses

Additional analyses were conducted to explore the role of paternal BMI. First, we presented the study population and the reproductive health outcomes across paternal BMI categorizations, as described above. Moreover, as described above for maternal BMI, we estimated associations with all reproductive health outcomes with paternal BMI as the exposure. Lastly, we additionally adjusted the main analysis for paternal BMI.

### Ethics

The data collected within the DNBC were approved by The Committee for Biomedical Research Ethics in Denmark ((KF)01-471/94) and in accordance with the Helsinki Declaration. The FEPOS cohort was approved by the Scientific Research Ethics Committee (No.: H-16015857), registered at the Danish Data Protection Agency (No.: 2012-58-0004), and approved by the steering committee of the DNBC (2018-09). Written informed consent was obtained from all participants upon participation.

## Results

In our study population, 64 (6%) were born by underweight mothers, 756 (73%) by normal weight mothers, 164 (16%) by overweight mothers, and 47 (5%) were born by mothers with pre-pregnancy obesity (Table 1). Underweight mothers were more likely to be themselves or to be with a partner that was a high-grade professional (37.5%) and to smoke in the first trimester (29.7%). Mothers with pre-pregnancy obesity were more likely to be themselves or to be with a partner who was a skilled or unskilled worker (55.3%), to drink alcohol in the first trimester (53.2%), and to have overweight or obese sons (36.2% and 10.6%, respectively). Moreover, sons of obese mothers were more likely to report an abstinence time of >3 days (46.8%) (Table 1).

The median sperm concentration in this population-based sample of young men (median age was 19 years, 1 months (pseudo range: 18 years, 9 months to 21 years, 4 months)) was 39 × 10^6^/ml (pseudo IQR: 19 × 10^6^/ml; 72 × 10^6^/ml). Sons of obese mothers had higher oestrogen, lower SHBG, and higher FAI than sons of non-obese mothers had. Surprisingly, they also had higher crude semen concentration and higher total sperm count (Table 2).

**Table 2.** Reproductive health outcomes* according to categorizations of maternal pre-pregnancy BMI in 1031 participants from the Fetal Programming of Semen Quality Cohort, Denmark, 1998–2019.

Categorical maternal pre-pregnancy BMI	Underweight	Normal weight	Overweight	Obese	Total
**N (%)**	64 (6.2)	756 (73.3)	164 (15.9)	47 (4.6)	1031 (100)
**Semen characteristics**					
Volume (ml)	2.4 (1.9; 3.3)	2.8 (2.0; 3.7)	2.5 (1.7; 3.4)	2.9 (1.5; 4.2)	2.7 (1.9; 3.7)
Concentration (mill/ml)	43 (20; 66)	38 (18; 74)	37 (23; 65)	47 (18; 89)	39 (19; 72)
Total sperm count (mill)	96 (35; 165)	104 (46; 206)	95 (45; 154)	171 (71; 247)	103 (46; 200)
Progressive motility (%)	63 (51; 73)	63 (52; 74)	63 (54; 74)	60 (48; 73)	63 (52; 74)
Morphology (% normal)	7.3 (3.7; 10.9)	6.0 (3.0; 10.0)	6.0 (3.3; 10.6)	7.0 (4.3; 11.3)	6.0 (3.0; 10.0)
DFI (%)	8.9 (6.0; 12.5)	10.0 (7.0; 14.0)	9.0 (6.0; 13.5)	9.6 (7.2; 12.1)	9.3 (7.0; 13.0)
HDS (%)	10.0 (6.8; 12.5)	9.0 (7.0; 13.0)	10.0 (7.0; 13.5)	8.2 (6.5; 11.9)	9.0 (7.0; 13.0)
**Testes volume**					
Average testes volume (ml)	15 (11; 20)	15 (12; 20)	15 (12; 20)	15 (11; 20)	15 (12; 20)
**Reproductive hormones**					
Testosterone (nmol/l)	19 (15; 24)	18 (15; 22)	17 (15; 21)	18 (15; 23)	18 (15; 22)
Oestradiol (pmol/l)	58 (33; 82)	51 (34; 72)	55 (38; 75)	63 (40; 80)	52 (35; 73)
SHBG (nmol/l)	32 (27; 47)	33 (26; 42)	32 (24; 40)	29 (22; 36)	33 (25; 41)
FSH (IU/l)	3.4 (2.8; 4.5)	3.6 (2.5; 5.2)	3.4 (2.3; 5.2)	3.3 (2.5; 4.6)	3.5 (2.5; 5.1)
LH (IU/l)	5.2 (4.1; 6.8)	5.1 (3.9; 6.5)	5.2 (4.0; 6.8)	5.1 (4.5; 6.7)	5.1 (4.0; 6.6)
FAI (%)	57 (45; 69)	55 (45; 68)	56 (47; 70)	65 (54; 79)	56 (45; 69)

DFI, DNA fragmentation index; HDS, high DNA stainability; SHBG, sex hormone-binding globulin; FAI, free androgen index.

* Reproductive health outcomes are presented as pseudo median values (pseudo interquartile range). A pseudo percentile is calculated as the average of the five percentiles nearest the actual percentile to comply with local data regulations.

Most reproductive hormone levels displayed a circadian rhythm, as expected. Levels of testosterone, oestrogen, LH, and FAI were highest in the morning and lowest in the evening. There were no fluctuations in SHBG and FSH across time of the day of blood sampling in our sample (Supplementary Table S1).

### Main analysis

Overall, we found no strong evidence of an association between pre-pregnancy BMI and semen characteristics or testes volume, with no clear pattern of an association (Table 3). However, there were some tendencies towards lower semen volume (−13% (95% CI: −24%; −1%) and lower DFI (−11% (95% CI: −23%; 1%) in sons born of underweight mothers, compared with sons of normal weight mothers. The linear regression models (Table 3) and the spline plots (Fig. 2) revealed a dose–response association between maternal pre-pregnancy BMI and higher oestradiol (overweight: 12% (95% CI: 1%; 25%), obese: 22% (95% CI: 5%; 43%)) and lower SHBG (overweight: −5% (95% CI: −11%; 2%), obese: −13% (95% CI: −24%; −1%)) in the sons. Sons of mothers with pre-pregnancy obesity further had higher LH (13% (95% CI: 2%; 26%)) and higher FAI (17% (95% CI: 7%; 29%)) compared with sons of normal weight mothers (Table 3). No strong patterns were observed for other hormone levels.

**Figure 2.**
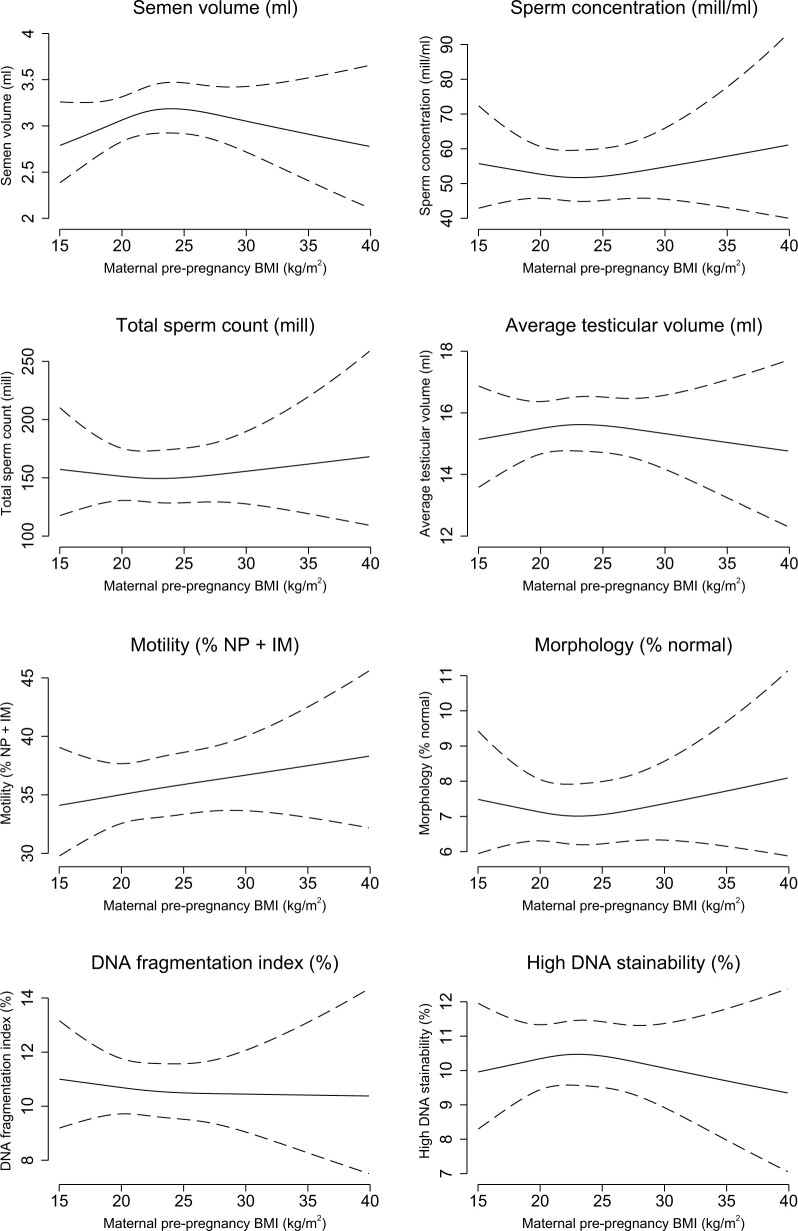
**Spline plots of the association between maternal pre-pregnancy BMI and semen characteristics, testes volume, and reproductive hormone levels in young adult sons.** Restricted cubic spline plots (with three knots at 10th percentile: 19.0 kg/m^2^, 50th percentile: 22.1 kg/m^2^, and 90th percentile: 27.3 kg/m^2^) of semen characteristics, testes volume, and reproductive hormone levels according to maternal pre-pregnancy BMI (solid lines) with 95% CI (dotted lines). The spline plots are presented for a reference son, whose parents' highest socioeconomic status was a high-grade professional, whose mother was 30 years of age at the delivery, was a non-smoker and abstained from any alcohol intake. The reference son had an abstinence time of 2.5 days, delivered his semen sample at the clinic, did not report any spillage of the semen sample, had his motility assessment performed 30 min after ejaculation, and had blood drawn for assessment of reproductive hormone levels between 12.00 and 18.00. NP, non-progressive motility; IM, immotile motility; SHBG, sex hormone-binding globulin.

**Figure 2.**
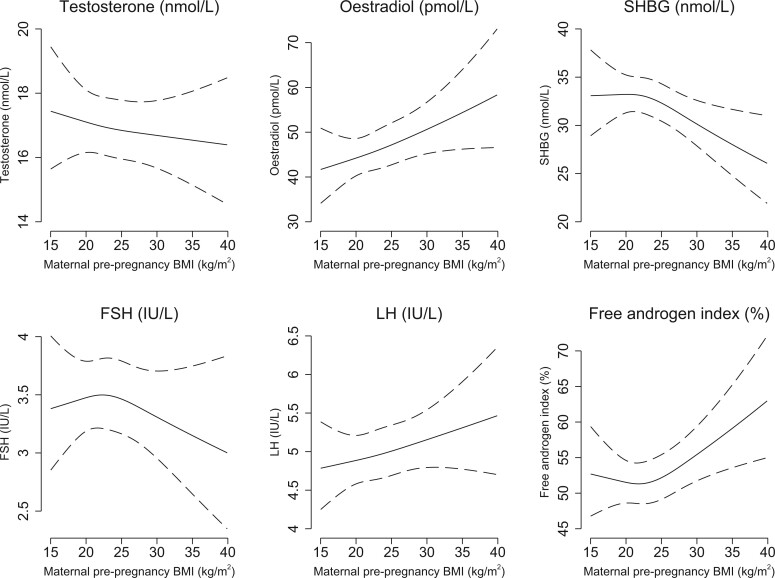
Continued.

**Table 3.** Relative differences in reproductive health outcomes in young adult sons according to categorizations of maternal pre-pregnancy BMI.

	Underweight		Overweight		Obese	
	Crude	**Adjusted** ^a^ **(95% CI)**	Crude	**Adjusted** ^a^ **(95% CI)**	Crude	**Adjusted** ^a^ **(95% CI)**
**Semen characteristics** ^b^						
Volume (ml)^c^	−13%	−13% (−24; −1)	−11%	−8% (−13; 1)	3%	1% (−16; 21)
Concentration (mill/ml)	−2%	8% (−12; 32)	−1%	4% (−10; 21)	13%	13% (−16; 52)
Total sperm count (mill)^c^	−3%	−4% (−25; 23)	−14%	−12% (−24; 3)	24%	21% (−7; 57)
Motility (NP + IM %)^d^	3%	−1% (−11; 9)	−1%	−2% (−9; 4)	9%	12% (0; 24)
Morphology (% normal)	10%	12% (−6; 34)	4%	9% (−4; 23)	13%	12% (−8; 36)
DFI (%)	−13%	−11% (−23; 1)	−5%	−4 (−12; 5)	2%	2% (−17; 27)
HDS (%)	1%	2% (−13; 19)	3%	1% (−8; 10)	−4%	−4% (−21; 17)
**Testes volume** ^e^						
Average testes volume (ml)	−4%	−4% (−13; 5)	−3%	−4% (−10; 1)	−4%	−2% (−13; 9)
**Reproductive hormones** ^f^						
Testosterone (nmol/l)	5%	4% (−4; 13)	−4%	−3% (−8; 2)	3%	2% (−6; 10)
Oestradiol (pmol/l)	7%	6% (−11; 27)	12%	12% (1; 25)	21%	22% (5; 43)
SHBG (nmol/l)	1%	−1% (−15; 14)	−5%	−5% (−11; 2)	−15%	−13% (−24; −1)
FSH (IU/l)	−3%	−6% (−18; 8)	−4%	−6% (−16; 5)	−5%	−5% (−20; 13)
LH (IU/l)	3%	2% (−8; 13)	4%	3% (−4; 11)	11%	13% (2; 26)
FAI (%)	4%	5% (−7; 20)	1%	2% (−4; 7)	20%	17% (7; 29)

Results are presented as relative percentage differences. Underweight, overweight, and obese relative to normal weight in 1031 participants from the Fetal Programming of Semen Quality (FEPOS) cohort, Denmark, 1998–2019.

NP, non-progressive motility; IM, immotile; DFI, DNA fragmentation index; HDS, high DNA stainability; SHBG, sex hormone-binding globulin; FAI, free androgen index.

a Adjusted for maternal age at delivery, highest parental social class, maternal first-trimester smoking, and alcohol intake.

b Further adjusted for abstinence time, spillage, and place of semen sample.

c Participants reporting spillage excluded.

d Further adjusted for from time from ejaculation to analysis.

e Further adjusted for abstinence time.

f Further adjusted for time of blood sample.

### Sensitivity analyses

In the sensitivity analysis of the semen characteristics excluding participants who collected the semen sample at home, results were overall comparable with the results from the main analysis (Supplementary Table S2). In the sensitivity analysis of the reproductive hormone levels excluding participants who had blood drawn in the evening, results attenuated slightly and led to the loss of statistical significance for the association between maternal pre-pregnancy obesity and SHBG (−11% (95% CI: −22%; 2%)) and LH (8% (95% CI: −3%; 21%)) (Supplementary Table S3). Not including abstinence time as a precision variable did not change the results considerably, however, we observed slightly higher total sperm count in sons of obese mothers relative to sons of normal weight mothers, compared with the main analysis (Supplementary Table S4). Fitting the models without inverse probability of selection weights gave similar results as the main analysis (Supplementary Table S5).

### Mediation analyses

Results from the mediation analysis investigating potential mediation by the sons’ birthweight suggested that the association between maternal pre-pregnancy BMI and the reproductive hormone levels was partly mediated by the sons’ birthweight (Supplementary Table S6). We did not observe any mediated effect of maternal pre-pregnancy BMI through the sons’ pubertal timing, indicating no mediation by the sons’ pubertal timing (Supplementary Table S7). Overall, the association between maternal pre-pregnancy BMI and reproductive hormone levels was partly mediated by the sons’ own fat mass (Supplementary Table S8) and the sons’ own BMI (Supplementary Table S9). However, most of the effect of maternal pre-pregnancy obesity on lower SHBG was mediated by the sons’ own fat mass (indirect effect: −11% (95% CI: −18%; −4%)) and the sons’ own BMI (indirect effect: −13% (95% CI: −20%; −5%)).

### Additional analyses

In total, 60% of the fathers of the sons in this study population were normal weight, 35% were overweight, and 5% were obese. Overweight and obese fathers were more likely to have overweight or obese sons, to be with overweight or obese mothers, and to be with mothers who smoked or had an alcohol intake in the first trimester. Normal weight fathers were more likely to be themselves or to be with a partner who was with high-grade professionals (Supplementary Table S10). Sons of obese fathers had lower testosterone, lower oestradiol, and lower SHBG than sons of non-obese fathers had. Surprisingly, they also had higher crude morphology (Supplementary Table S11).

When analysing the associations between paternal BMI and reproductive health outcomes while adjusting for the potential confounding factors in addition to maternal pre-pregnancy BMI, we did not observe any consistent findings for either semen characteristics, testes volume, or reproductive hormone levels (Supplementary Table S12). Lastly, further adjusting the main analyses for paternal BMI did not alter the overall associations between maternal pre-pregnancy BMI and reproductive health outcomes (Supplementary Table S13).

## Discussion

### Main findings

In this large study of >1000 young men, we found a low prevalence of overweight and obesity in both mothers and sons. The sons had overall low sperm concentration with a median sperm concentration of 39 × 10^6^/ml; a level at which fecundity might be impaired, as a sperm concentration below 40 × 10^6^/ml has been associated with prolonged time to pregnancy (Bonde *et al.*, 1998). This compares with other population-based samples of young Danish men; e.g. a sperm concentration of 44 × 10^6^/ml have been found in Danish conscripts (Priskorn *et al.*, 2018), and in the pilot study of 328 young Danish men, a sperm concentration of 37 × 10^6^/ml was found (Ramlau-Hansen *et al.*, 2007).

We found no consistent associations between maternal pre-pregnancy BMI and semen characteristics or testes volume. Sons of mothers with pre-pregnancy overweight or obesity presented with an altered reproductive hormonal profile characterized by higher levels of oestradiol, LH, and FAI, and lower levels of SHBG. These results were robust across several sensitivity analyses. Whereas the associations with higher oestradiol, LH, and FAI were partly mediated by the sons’ birthweight, their own fat mass, and BMI, most of the effect on lower SHBG in sons of overweight and obese mothers was mediated by the sons’ own fat mass and BMI. Additional analyses investigating paternal BMI suggested that this was not a strong confounder in this study.

### Strengths and limitations

The main strengths of this study were the longitudinal design with the assessment of maternal pre-pregnancy BMI and potential confounding factors in early pregnancy and detailed assessment of many reproductive health outcomes in a large sample of young men from the general population 19 years later.

Though we studied a large sample of young men, the participation rate was low. Even though most baseline parental characteristics were associated with participation, in particular alcohol intake and social class, we have previously found limited risk of selection bias in a validation study examining the risk of potential selection bias in the FEPOS cohort (Gaml-Sørensen *et al.*, 2023). The participants were young and likely unaware of their semen quality or hormone levels when consenting to participate. Moreover, maternal pre-pregnancy BMI was not associated with participation. We estimated selection weights and employed these in the statistical models, thereby further limiting the risk of selection bias. When fitting all models without the selection weights the results did not change (Supplementary Table S5) and the risk of selection bias in this study is therefore considered low.

Information on maternal pre-pregnancy BMI was obtained by self-report in early pregnancy. This self-reporting may have introduced some misclassification due to recall. Moreover, self-reporting of height and weight may not be as accurate as height and weight measurements carried out by trained clinicians. However, a systematic review and meta-analysis found that the magnitude of any under- or over-estimation in women of reproductive age was minor and concluded that self-reported height and weight were applicable in research settings (Seijo *et al.*, 2018). Moreover, the validity of the self-reported weights measures has been validated in the DNBC in a sample of 5033 women, revealing limited misclassification (Nøhr and Nøhr, 2005). Any misclassification is likely non-differential with regard to the outcomes under study, probably leading to bias towards the null.

The assessment of semen characteristics was performed using a state-of-the-art laboratory set-up that was continuously quality controlled (Keglberg Hærvig *et al.*, 2020). Though participants were instructed to maintain a minimum of 2–3 days of sexual abstinence, around one-third of the participants did not comply with this instruction; however, we adjusted all analyses investigating semen characteristics for actual abstinence time. We observed higher total sperm count in sons of obese mothers relative to sons of normal weight mothers, when we excluded abstinence time as a precision variable, which may be explained by the longer abstinence time observed in sons of obese mothers (Supplementary Table S4). Overall, however, the results when excluding abstinence time were comparable to the main analysis. Some participants (∼13%) collected the semen sample at home, therefore time from ejaculation to analysis was delayed for those participants (253 participants had their semen sample analysed >1 h after ejaculation). We accounted for this by adjusting all analyses investigating semen characteristics for place of semen sample collection. All analyses investigating motility was in addition also adjusted for time from ejaculation to analysis. In the sensitivity analysis excluding the semen samples that were collected at home, the results remained overall similar, highlighting the robustness of the results (Supplementary Table S2).

The laboratory technicians performing the assessment were blinded to the sons' exposure status, and any measurement errors are most likely non-differential regarding maternal pre-pregnancy BMI. The sons only provided one semen sample for analysis, and the within individual variation in semen characteristics may introduce additional error. However, previous studies have found that this does not introduce bias in associational studies (Stokes-Riner *et al.*, 2007; Hart *et al.*, 2015). Testes volume was self-measured by the participants using a Prader Orchidometer. Though this method poses a risk of underestimation, self-assessment of testes volume using this method has been shown to be valid compared with an expert examiner (Ramlau-Hansen *et al.*, 2007), and any underestimation is likely non-differential with regards to maternal pre-pregnancy BMI. Assessment of reproductive hormone levels may also induce some measurement errors, also likely non-differential with regard to maternal pre-pregnancy BMI. The daily fluctuations in reproductive hormones were handled by adjusting for the time of the day of blood sampling. In the sensitivity analysis excluding the participants who provided a blood sample for reproductive hormone assessment during the evening, some estimates attenuated slightly, leading to a loss of statistical significance for the association between maternal pre-pregnancy obesity and SHBG and LH. However, most results were still overall comparable with the results from the main analysis (Supplementary Table S3).

Though we adjusted for several important potential confounding factors, this observational study cannot provide causal estimates owing to the inherent risk of residual confounding. Paternal periconceptional characteristics, including paternal BMI may also be important for later health in the sons (Carter *et al.*, 2023) and may potentially be an important confounding factor, when studying maternal pre-pregnancy BMI. However, the additional analyses did not suggest paternal BMI to be a strong confounding factor in this study. There may, however, still be some unmeasured shared characteristics influencing both the maternal BMI prior to pregnancy and the reproductive health outcomes in the adult sons; therefore, the causal effect of maternal BMI should be interpreted with caution. Also, we cannot exclude the risk of type-1 errors owing to the multiple testing.

### Interpretation

In a pilot study of 328 young Danish men, Ramlau-Hansen *et al.* (2007) found that crude sperm concentration and inhibin B tended to be lower in sons of mothers with pre-pregnancy overweight/obesity compared with sons of mothers with normal pre-pregnancy BMI. The associations were small and only suggestive of an effect, and the study did not distinguish between maternal pre-pregnancy overweight and obesity because few mothers were overweight or obese (Ramlau-Hansen *et al.*, 2007). We had a larger population and sufficient power to differentiate between overweight and obesity; however, no associations with semen characteristics or testes volume were observed, in line with the results from the previous study.

In our study, we found associations with reproductive hormone levels only and not with semen characteristics or testes volume. It is possible that the participants, because of their young age at inclusion to this study, possess highly robust semen characteristics, likely not to be affected by the altered reproductive hormone profile. However, this finding may also be explained by the low variability across the semen characteristics in this cohort of young men from the general population, since this may reduce the statistical power to detect an association between maternal pre-pregnancy BMI and the reproductive health outcomes.

Although we did not observe strong associations in our study, other studies support that maternal overweight or obesity might affect the development of the male reproductive system. Pre-pregnancy obesity has been associated with an increased risk in male offspring of cryptorchidism and hypospadias (Arendt *et al.*, 2017), earlier pubertal timing (Brix *et al.*, 2019), and increased odds of infertility (Arendt *et al.*, 2021).

Different plausible pathways are hypothesized to link higher maternal pre-pregnancy BMI to male-offspring reproductive health. Maternal pre-pregnancy overweight or obesity may affect the intrauterine environment and foetal development, potentially through elevated adipose tissue, hyperglycaemia, and foetal hyperinsulinemia and be associated with a broad spectrum of diverse metabolic and reproductive health outcomes (HAPO Study Cooperative Research Group, 2009; Hemond *et al.*, 2016). Fat is a hormonally active tissue, and adiposity is associated with higher levels of free oestradiol through reduced levels of SHBG (Botwood *et al.*, 1995; Pettigrew and Hamilton-Fairley, 1997; Pasquali, 2006). Exposure to high levels of oestrogens during foetal life may in turn play a role in determining the function of male reproductive health in adult life (Sharpe and Skakkebaek, 1993). This hypothesis remains elusive, however, and a shift towards a focus on environmental factors has emerged (Skakkebæk *et al.*, 2022). In addition, fat tissue may also accumulate endocrine-disrupting chemicals that may have the potential to harm male reproductive health (Le Magueresse-Battistoni, 2020).

We did several mediation analyses to investigate plausible mechanisms in the association between maternal pre-pregnancy BMI and male reproductive health: that is the potential pathway through the sons’ birthweight, pubertal timing in addition to their own fat mass, and BMI. We found that the association between maternal pre-pregnancy BMI and reproductive hormone levels was partly mediated by the sons’ birthweight; however, researchers should keep in mind the risk of introducing collider-stratification bias when interpreting the results. Though we did not observe any mediation by the sons’ pubertal timing, we cannot eliminate that maternal pre-pregnancy BMI in fact may be associated with reproductive hormone levels through pubertal timing (Brix *et al.*, 2019; Brix *et al.*, 2023); however, measurement errors in the composite marker of pubertal timing may underestimate the potential mediating effect of altered pubertal timing on the reproductive hormone levels. Since maternal pre-pregnancy BMI may determine the adult sons’ own fat mass and BMI (Tequeanes *et al.*, 2009), and since overweight and obesity in adult males may be associated with low fecundity, potentially owing to associations with lower levels of testosterone and SHBG and higher levels of oestradiol (Salas-Huetos *et al.*, 2021), we examined the potential mediating role of sons’ fat mass and BMI. We observed higher levels of oestradiol and lower levels of SHBG in sons born of mothers with pre-pregnancy overweight and obesity, and these associations were partly mediated by the sons’ own fat mass and BMI. This suggests that some of the potential consequences for reproductive health of being born by mothers with higher pre-pregnancy BMI may be reduced by introducing preventative actions targeting the increased fat mass and BMI in adult sons.

In this study, we observed a relative low prevalence of overweight and obesity among both mothers and sons. Therefore, comparing these results to other populations characterized by higher a prevalence of overweight and obesity among mothers and sons should be undertaken cautiously.

## Conclusion

We did not find associations between maternal pre-pregnancy BMI and semen characteristics and testes volume in adult sons. We observed an altered reproductive hormone profile, characterized by higher levels of oestradiol, LH, and FAI, and lower levels of SHBG in sons born of mothers with pre-pregnancy overweight and obesity. The potential consequences of this altered reproductive hormone profile on reproductive health or other health outcomes remain to be investigated. Since the associations were partly mediated by the sons' own fat mass and BMI, the potential consequences of maternal pre-pregnancy obesity on the son's reproductive hormone profile may be mitigated through approaches aimed at reducing adult overweight and obesity in the sons.

## Supplementary Material

dead230_Supplementary_Data_File_S1

dead230_Supplementary_Figure_S1

dead230_Supplementary_Table_S1

dead230_Supplementary_Table_S2

dead230_Supplementary_Table_S3

dead230_Supplementary_Table_S4

dead230_Supplementary_Table_S5

dead230_Supplementary_Table_S6

dead230_Supplementary_Table_S7

dead230_Supplementary_Table_S8

dead230_Supplementary_Table_S9

dead230_Supplementary_Table_S10

dead230_Supplementary_Table_S11

dead230_Supplementary_Table_S12

dead230_Supplementary_Table_S13

## Data Availability

The dataset analysed in the study is not publicly available due to national data security legislation on sensitive personal data. Researchers may apply for access to data from the DNBC. Please see https://www.dnbc.dk/data-available for additional information.
