# Supplementary material for: Maternal pre-pregnancy BMI and reproductive health in adult sons: a study in the Danish National Birth Cohort
Source: Hum Reprod. 2023 Nov 4;39(1):219–31. doi: 10.1093/humrep/dead230 (PMC10767916; doi:10.1093/humrep/dead230)
Supplement: dead230_Supplementary_Data_File_S1 [file dead230_supplementary_data_file_s1.docx]

**Supplementary Data File S1**

**Checklist from ‘How to count sperm properly’: checklist for acceptability of studies. Björndahl et al., Hum Reprod 2016, 31: 227-232**

**A check list for authors, reviewers and editors**

Any deviation from this guideline (i.e. a box without a tick) must be declared and explained in the Materials and Methods section of the manuscript, including explaining its effect on the measurement uncertainty of the data, in order to allow the reader to evaluate the quality of the analyses performed. Investigations that would be subject to this requirement can roughly be classified as clinical (evaluating patient treatment, diagnostic classification or predictive powers of certain assessments), experimental (e.g. exposure of sperm to different compounds or *in vitro* treatments ([Mortimer *et al.*, 2013](javascript:;))), or epidemiological (evaluating variations in semen characteristics or effects of exposure populations to certain compounds or other circumstances). Any scientific rationale for not complying with the guidelines, which is not included in the Materials and Methods section of the manuscript, must be substantiated to Editor and reviewers.

**Patients**

- **X** For clinical studies: The patient population (e.g. patients, volunteers, students) has been declared in the manuscript, together with the recruitment method and inclusion and exclusion criteria. If the study concerns couples being investigated for infertility then the following must be specified in the manuscript: fertility status of female partner; and primary, secondary or other level of investigation of the man.
- **N/A** If used in the manuscript, the term ‘male factor’ must be completely defined.

**General aspects**

- □ Patients were instructed to maintain 2–7 days of sexual abstinence before collecting a sample for investigation. *Comment:* *The participants were instructed to maintain a minimum of 2-3 days of sexual abstinence. For the entire FEPOS cohort, in total n=366 participants maintained less than 2 days of sexual abstinence; n=340 participants maintained the recommended 2-3 days of sexual abstinence; and n=347 participants maintained more than 3 days of sexual abstinence before collecting the semen sample for investigation. Abstinence time was recorded at the clinical visit and taken into account all analyses.*
- **X**  Patients were informed about the importance of reporting any missed early ejaculate fractions, and men's answers were noted on the laboratory form.
- **X**  For specimens not collected at the laboratory, patients were instructed to avoid cooling or heating of the semen sample during transport to the laboratory.
- **X**  Samples were kept at 37°C before initiation of and during the analysis in case of sperm motility assessment.
- □ For samples collected adjacent to the laboratory, analysis was initiated after completion of liquefaction and within 30 min after ejaculation. If this was not done—and more importantly when some of the samples are collected in the laboratory and others are collected at home—it should be checked that this did not influence the data (and, if yes, that this effect must be included as a confounding factor in the statistical analysis). *Comment: Participants in FEPOS collected the semen sample either at home (n=138) or at the clinic (n=910). Though semen sample collection at the clinics were encouraged, participants living within an hour drive from the clinic had the opportunity to collect the semen sample at home. All analyses was adjusted for place at semen sample collection (at home or in the clinic).*

*Time from ejaculation to analysis in minutes was recorded at the clinical visit regardless of place of semen sample collection. For the entire FEPOS cohort, in total n=35 semen samples were analysed before 30 min; n=751 samples were analysed between 30-60 min; n=260 semen samples were analysed after 60 min. Time from ejaculation to analysis was taken into account in analyses investigating total motility.*

- **X**  Liquefaction was first checked within 30 min after ejaculation.
- **X**  Volume was determined either by weighing or using a wide-bore volumetric pipette.
- □ Viscosity was measured using either a wide-bore pipette or a glass rod. *Comment: Viscosity was not measured, rather it was visually assessed as normal or abnormal.*
- **X**  All staff members who performed the analyses have been trained in basic semen analysis (ESHRE Basic Semen Analysis Course—or equivalent—and further in-house training) and participate regularly in internal quality control.
- **X**  If more than one method can be recommended for a particular characteristic (e.g. to measure volume), only one should be used in a given study.

**Sperm concentration assessment**

- **X**  Semen aliquot to be diluted for sperm concentration assessment was taken with a positive displacement pipette (i.e. a ‘PCR pipette’) using a recommended diluent (state which diluent: NaHCO_3_).
- □ Only standard dilutions were used (1:50, 1:20, or 1:10). *Comment: Standard dilutions were used where applicable; however, in very few cases 1:1, 1:2, 1:5 and 1:14 was used.*
- **X**  Sperm concentration was assessed using haemocytometers with improved Neubauer ruling.
- **X**  Haemocytometers were allowed to rest for 10–15 min in a humid chamber to allow sedimentation of the suspended spermatozoa onto the counting grid before counting.
- **X**  Sperm counting was done using phase contrast microscope optics (200–400×).
- **X**  Comparisons were made between duplicate counts, and counts re-done when the difference exceeded the acceptance limits.
- **X**  Typically at least 200 spermatozoa were counted in each of the duplicate assessments.

**Sperm motility assessment**

- **X**  Motility assessments were performed at 37°C ± 0.5°C.
- **X**  Motility assessments were done using phase contrast microscope optics (200–400×).
- □ Sperm motility was classified using a four-category scheme: rapid progressive, slow progressive, non-progressive, and immotile (World Health Organization, 1999; Björndahl *et al.*, 2010; Barratt *et al.*, 2011). *Comment:* *The motility was assessed as progressive spermatozoa (a), non-progressive spermatozoa (b) and immotile (c) as recommended in* *WHO laboratory manual for the Examination and processing of human semen, fifth edition (WHO laboratory manual for the examination and processing of human semen, 2010). The outcome used in the study was progressive (a) motility, which, due to model fit, was assessed as non-progressive spermatozoa (b) + immotile (c) motility.*
- **X**  Motility assessments were done in duplicate and compared; counts were re-done on new preparations when the difference between duplicates exceeded the acceptance limits.
- **X**  The wet preparation was made with a drop of 6 µl and a 18x18 mm coverslip to give a depth of 18.5 µm (must be at least 10 µm, but not too deep so as to allow spermatozoa to move freely in and out of focus; typically *ca.* 20 µm).
- **X**  At least 200 spermatozoa were assessed in each duplicate motility count.
- **X**  At least 5 microscope fields of view were examined in each duplicate count.

**Sperm vitality assessment** *Comment:* *Sperm vitality assessment was not done in FEPOS.*

- **N/A** A validated supravital staining, appropriate to the type of microscope optics utilized, was used to assess sperm vitality.
- **N/A** At least 200 spermatozoa were evaluated in each sample.
- **N/A** Assessments were done under high magnification (×1000–1250) using a 100× high resolution oil immersion objective and bright field microscope optics (Köhler illumination).

**Sperm morphology assessment**

 □ Tygerberg Strict Criteria were used for the evaluation of human sperm morphology. *Comment:* *Sperm morphology assessments was done according to WHO laboratory manual for the Examination and processing of human semen, fifth edition (WHO laboratory manual for the examination and processing of human semen, 2010).*

Note: Another classification could be used for scientific studies with specific aims if the classification is described or referenced. Depending on the aim of the study, the evaluation of particular abnormal forms might be useful.

- **X**  Abnormalities are recorded for all four regions of the spermatozoon (head, neck/midpiece, tail and cytoplasmic residue) and the Teratozoospermia Index or ‘TZI’ was calculated (Björndahl *et al.*, 2010; Barratt *et al.*, 2011).
- □ If the laboratory claims to use Tygerberg Strict Criteria for the evaluation of human sperm morphology, then the laboratory must participate in an external quality assurance scheme to verify that its assessments comply with these criteria. *Comment: The Centre of Reproductive Medicine in Malmö, Sweden, where the morphology assessments were done, not only participates in external quality control; it acts as a reference laboratory for ESHRE-NAFA (Nordic Association of Andrology) External Quality Control.*
- **X**  The Papanicolaou staining method adapted for the assessment of human sperm morphology was used. For specific aims other staining methods could be used, but must then be declared and explained.
- **X**  At least 200 spermatozoa were assessed in each ejaculate.
- **X**  Assessments were done under high magnification (×1000–1250) using a 100× high resolution oil immersion objective and bright field microscope optics (Köhler illumination).

**Other findings**

- **X**  The presence of abnormal clumping (aggregates and agglutinates) was recorded.
- **X**  Abnormal viscosity was recorded.
- **X**  The presence of inflammatory cells was recorded and reported if more than 1 million/ml.
- **N/A** For the purpose of classifying infertility status (World Health Organization, 2010), antisperm antibodies were examined with a validated screening test (state which method was used: ________________).

**Analysing data**

- **X**  The actual duration of sexual abstinence (in ‘hours’ or ‘days’) was recorded for each sample and included in the data reported in the manuscript.
- **X**  As a minimum in clinical studies, semen volume, sperm concentration, total number of spermatozoa/ejaculate, and abstinence time are given to reflect sperm production and output; only samples identified as having been collected completely can be included in the study.
- **X**  Confounding factors have been considered for statistical analysis: e.g. abstinence time and age, to evidence secular or geographical variations in sperm concentration or sperm count.
- **N/A** If appropriate, optional biochemical markers for prostatic, seminal vesicular and epididymal secretions were analysed and reported both as concentration and total amount.
- □ Signs of active infection/inflammation were noted and considered in the analysis of data in the study (e.g. inflammatory cells, impaired sperm motility, possibly also antisperm antibodies and reduction of secretory contributions). *Comment:* *Signs of active inflammation was noted; however, the proportion of samples with the presence of inflammatory cells > 1 mill/ml was 0.7% and therefore, too small to consider in the analyses.*

**Reference**

*WHO laboratory manual for the examination and processing of human semen*. (2010). (5. ed. ed.). Geneva: World Health Organization.
