# Supplementary material for: Maternal pre-pregnancy BMI and reproductive health in adult sons: a study in the Danish National Birth Cohort
Source: Hum Reprod. 2023 Nov 4;39(1):219–31. doi: 10.1093/humrep/dead230 (PMC10767916; doi:10.1093/humrep/dead230)
Supplement: dead230_Supplementary_Figure_S1 [file dead230_supplementary_figure_s1.pdf]

Human Reproduction, 2023, 1–1
https://doi.org/10.1093/humrep/dead230
Supplementary Data

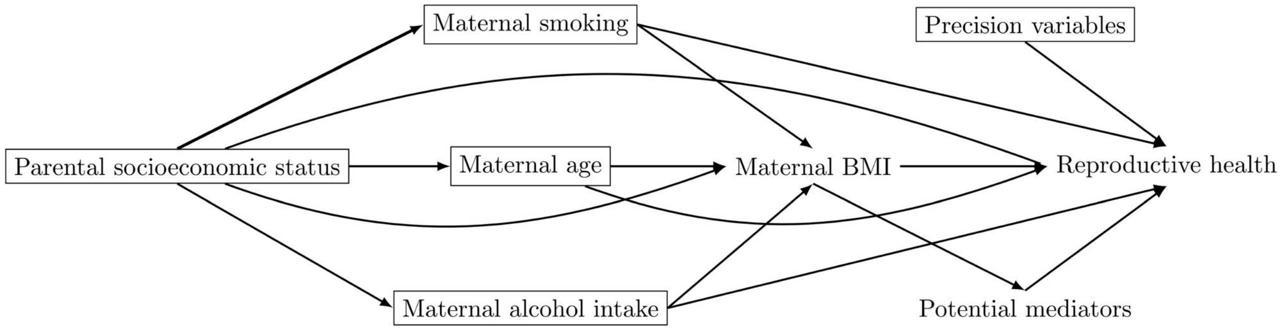

Supplementary Figure S1. Directed acyclic graph of the underlying framework of the study. Maternal pre-pregnancy BMI and reproductive health in adult sons: a study in the Danish National Birth Cohort. Directed acyclic graphs (DAGs) were used to identify potential confounding variables. Boxes indicate conditioning in the statistical analyses. Potential mediators include birthweight, pubertal timing, fat mass, and BMI.
