# Supplementary material for: Maternal pre-pregnancy BMI and reproductive health in adult sons: a study in the Danish National Birth Cohort
Source: Hum Reprod. 2023 Nov 4;39(1):219–31. doi: 10.1093/humrep/dead230 (PMC10767916; doi:10.1093/humrep/dead230)
Supplement: dead230_Supplementary_Table_S1 [file dead230_supplementary_table_s1.pdf]

OXFORD
human reproduction

Human Reproduction, 2023, 1–1
https://doi.org/10.1093/humrep/dead230
Supplementary Data

Supplementary Table S1. Reproductive hormone levels* according to time of blood sampling in participants from the Fetal Programming of Semen Quality Cohort, Denmark, 1998–2019.

Time of blood sampling	Morning (before 12.00)	Afternoon (12.00–18.00)	Evening (after 18.00)	P-value
Testosterone (nmol/l)	19.5 (16.1; 23.0)	17.5 (14.4; 22.0)	15.9 (12.4; 19.6)	0.00
Oestradiol (pmol/l)	53.8 (36.3; 73.8)	51.8 (34.6; 72.3)	50.4 (24.9; 72.6)	0.15
SHBG (nmol/l)	32.6 (25.0; 42.0)	33.0 (26.0; 41.0)	33.7 (24.8; 41.0)	0.97
FSH (IU/l)	3.5 (2.5; 5.0)	3.5 (2.4; 5.2)	3.5 (2.6; 5.3)	0.84
LH (IU/l)	5.3 (4.2; 6.6)	4.9 (3.9; 6.5)	4.8 (3.6; 6.7)	0.02
FAI (%)	59.7 (50.0; 72.7)	54.2 (43.5; 66.6)	48.1 (37.7; 58.9)	0.00

*Reproductive hormone levels are presented as pseudo median values (pseudo interquartile range). A pseudo percentile is calculated as the average of the five percentiles nearest the actual percentile to comply with local data regulations.
SHBG, sex hormone-binding globulin; FAI, free androgen index.
