# Supplementary material for: Maternal pre-pregnancy BMI and reproductive health in adult sons: a study in the Danish National Birth Cohort
Source: Hum Reprod. 2023 Nov 4;39(1):219–31. doi: 10.1093/humrep/dead230 (PMC10767916; doi:10.1093/humrep/dead230)
Supplement: dead230_Supplementary_Table_S3 [file dead230_supplementary_table_s3.pdf]

OXFORD

human reproduction

Human Reproduction, 2023, 1–1
https://doi.org/10.1093/humrep/dead230
Supplementary Data

Supplementary Table S3. Relative differences in reproductive hormone levels in young adult sons according to categorizations of maternal pre-pregnancy BMI.

	Underweight		Overweight		Obese	
	**Crude**	**Adjusted[a] (95% CI)**	**Crude**	**Adjusted[a] (95% CI)**	**Crude**	**Adjusted[a] (95% CI)**
**Reproductive hormones[b]**						
Testosterone (nmol/l)	3%	2% (−6; 11)	−4%	−3% (−9; 2)	4%	3% (−5; 12)
Oestradiol (pmol/l)	7%	6% (−11; 28)	11%	12% (1; 25)	20%	23% (5; 44)
SHBG (nmol/l)	0%	−3% (−16; 13)	−4%	−4% (−10; 3)	−12%	−11% (−22; 2)
FSH (IU/l)	−2%	−5% (−18; 10)	−5%	−7% (−17; 4)	−10%	−5% (−19; 10)
LH (IU/l)	1%	0% (−10; 11)	2%	1% (−6; 8)	7%	8% (−3; 21)
FAI (%)	2%	5% (−8; 20)	0%	0% (−5; 6)	17%	16% (5; 27)

Results are presented as relative percentage differences. Underweight, overweight, and obese relative to normal weight in participants from the Fetal Programming of Semen Quality (FEPOS) cohort, Denmark, 1998–2019. Participants, who had blood drawn during the evening, were excluded from the analyses. SHBG, sex hormone-binding globulin; FAI, free androgen index.

[a] Adjusted for maternal age at delivery, highest parental social class, maternal first-trimester smoking and alcohol intake, and time of blood sample.

[b] Further adjusted for time of blood sample.
