# Supplementary material for: Maternal pre-pregnancy BMI and reproductive health in adult sons: a study in the Danish National Birth Cohort
Source: Hum Reprod. 2023 Nov 4;39(1):219–31. doi: 10.1093/humrep/dead230 (PMC10767916; doi:10.1093/humrep/dead230)
Supplement: dead230_Supplementary_Table_S4 [file dead230_supplementary_table_s4.pdf]

OXFORD

human reproduction

Human Reproduction, 2023, 1–1
https://doi.org/10.1093/humrep/dead230
Supplementary Data

Supplementary Table S4. Relative differences in semen characteristics in young adult sons according to categorizations of maternal pre-pregnancy BMI.

	Underweight		Overweight		Obese	
	Crude	Adjusted[a] (95% CI)	Crude	Adjusted[a] (95% CI)	Crude	Adjusted[a] (95% CI)
**Semen characteristics**						
Volume (ml)[b]	−14%	−16% (−27; −3)	−11%	−9% (−17; 0)	7%	5% (−11; 25)
Concentration (mill/ml)	−2%	3% (−18; 30)	−7%	1% (−; 17)	14%	18% (−10; 54)
Total sperm count (mill)[b]	−6%	−3% (−31; 37)	−22%	−14% (−28; 3)	28%	29% (1; 65)
Motility (NP + IM %)[c]	1%	−1% (−10; 10)	−2%	−2% (−8; 5)	9%	11% (0; 24)
Morphology (% normal)	10%	11% (−7; 32)	8%	8% (−5; 22)	17%	13% (−7; 37)
DFI (%)	−13%	−14% (−25; −1)	−7%	−6% (−15; 4)	6%	5% (−15; 28)
HDS (%)	0%	2% (−13; 20)	3%	1% (−7; 10)	−7%	−5% (−22; 16)

Results are presented as relative percentage differences. Underweight, overweight, and obese relative to normal weight in participants from the Fetal Programming of Semen Quality (FEPOS) cohort, Denmark, 1998–2019, without adjustment for abstinence time.
NP, non-progressive motility; IM, immotile; DFI, DNA fragmentation index; HDS, high DNA stainability.

[a] Adjusted for maternal age at delivery, highest parental social class, maternal first-trimester smoking, alcohol intake, place of semen sample, and spillage.
[b] Participants reporting spillage excluded.
[c] Further adjusted for time from ejaculation to analysis.
