# Supplementary material for: Maternal pre-pregnancy BMI and reproductive health in adult sons: a study in the Danish National Birth Cohort
Source: Hum Reprod. 2023 Nov 4;39(1):219–31. doi: 10.1093/humrep/dead230 (PMC10767916; doi:10.1093/humrep/dead230)
Supplement: dead230_Supplementary_Table_S5 [file dead230_supplementary_table_s5.pdf]

OXFORD

human reproduction

Human Reproduction, 2023, 1–1

https://doi.org/10.1093/humrep/dead230

Supplementary Data

Supplementary Table S5. Relative differences in semen characteristics in young adult sons according to categorizations of maternal pre-pregnancy BMI.

	Underweight		Overweight		Obese	
	Crude	Adjusted[a] (95% CI)	Crude	Adjusted[a] (95% CI)	Crude	Adjusted[a] (95% CI)
**Semen characteristics**[b]						
Volume (ml)[c]	−13%	−10% (−21; −1)	−11%	−9% (−16; −1)	3%	−1% (−16; 18)
Concentration (mill/ml)	−2%	5% (−13; 27)	−1%	2% (−12; 17)	13%	14% (−14; 51)
Total sperm count (mill)[c]	−3%	−3% (−26; 27)	−14%	−13% (−27; 2)	24%	21% (−10; 64)
Motility (NP + IM %)[d]	3%	−1% (−10; 9)	−1%	−2% (−8; 5)	9%	11% (−1; 24)
Morphology (% normal)	10%	12% (−5; 33)	4%	6% (−6; 19)	13%	14% (−5; 36)
DFI (%)	−13%	−11% (−21; 1)	−5%	−4 (−13; 5)	2%	−1% (−18; 20)
HDS (%)	1%	0% (−14; 16)	3%	3% (−5; 12)	−4%	−4% (−20; 15)
**Testes volume**[e]						
Average testes volume (ml)	−4%	−4% (−12; 5)	−3%	−3% (−9; 3)	−4%	−3% (−13; 8)
**Reproductive hormones**[f]						
Testosterone (nmol/l)	5%	5% (−3; 13)	−5%	−3% (−8; 2)	2%	1% (−7; 10)
Oestradiol (pmol/l)	8%	7% (−10; 27)	9%	13% (2; 26)	13%	23% (5; 45)
SHBG (nmol/l)	1%	1% (−11; 14)	−6%	−5% (−10; 1)	−14%	−14% (−24; −3)
FSH (IU/l)	−7%	−3% (−15; 10)	−2%	−3% (−14; 7)	−9%	−5% (−19; 10)
LH (IU/l)	2%	3% (−7; 13)	5%	5% (−2; 13)	8%	12% (1; 23)
FAI (%)	15%	4% (−7; 16)	0%	2% (−4; 7)	18%	18% (7; 29)

Results are presented as relative percentage differences. Underweight, overweight, and obese relative to normal weight in participants from the Fetal Programming of Semen Quality (FEPOS) cohort, Denmark, 1998–2019, without applying inverse probability of selection weights (IPSW) to the regression models. NP, non-progressive motility; IM, immotile; DFI, DNA fragmentation index; HDS, high DNA stainability; SHBG, sex hormone-binding globulin; FAI, free androgen index.

[a] Adjusted for maternal age at delivery, highest parental social class, maternal first-trimester smoking, and alcohol intake.
[b] Further adjusted for abstinence time, spillage, and place of semen sample.
[c] Participants reporting spillage excluded.
[d] Further adjusted for from time from ejaculation to analysis.
[e] Further adjusted for abstinence time.
[f] Further adjusted for time of blood sample.
