# Supplementary material for: Maternal pre-pregnancy BMI and reproductive health in adult sons: a study in the Danish National Birth Cohort
Source: Hum Reprod. 2023 Nov 4;39(1):219–31. doi: 10.1093/humrep/dead230 (PMC10767916; doi:10.1093/humrep/dead230)
Supplement: dead230_Supplementary_Table_S6 [file dead230_supplementary_table_s6.pdf]

Human Reproduction, 2023, 1–1
https://doi.org/10.1093/humrep/dead230
Supplementary Data

Supplementary Table S6. Mediation analysis investigating the potential mediating effect of sons' birthweight on the association between maternal pre-pregnancy BMI and reproductive health outcomes.

	Underweight		Overweight		Obese	
	Direct effect (NDE) adjusted[a] (95% CI)	Indirect effect (NIE) adjusted[a] (95% CI)	Direct effect (NDE) adjusted[a] (95% CI)	Indirect effect (NIE) adjusted[a] (95% CI)	Direct effect (NDE) adjusted[a] (95% CI)	Indirect effect (NIE) adjusted[a] (95% CI)
**Semen characteristics**[b]						
Volume (ml)[c]	−10% (−31; 16)	0% (−12; 12)	−10% (−20; 2)	−1% (−4; 2)	1% (−19; 26)	−2% (−13; 11)
Concentration (mill/ml)	0% (−22; 29)	6% (−7; 21)	2% (−13; 19)	−1% (−4; 3)	31% (−4; 78)	−13% (−25; 0)
Total sperm count (mill)[c]	9% (−27; 62)	−3% (−19; 15)	−12% (−26; 6)	−4% (−8; 0)	33% (−6; 88)	−11% (−24; 5)
Motility (NP + IM %)[d]	0% (−13; 14)	1% (−6; 8)	−1% (−8; 6)	0% (−2; 2)	13% (−2; 29)	−1% (−8; 6)
Morphology (% normal)	15% (−11; 48)	−2% (−13; 11)	2% (−10; 17)	4% (0; 8)	19% (−8; 54)	−3% (−15; 9)
DFI (%)	−7% (−22; 10)	−2% (−10; 8)	−5% (−14; 4)	−1% (−3; 2)	2% (−14; 22)	−2% (−11; 7)
HDS (%)	−6% (−19; 10)	5% (−4; 15)	2% (−7; 11)	1% (−2; 3)	−1% (−17; 18)	−4% (−12; 6)
**Testes volume**[e]						
Average testes volume (ml)	−7% (−17; 3)	2% (−3; 8)	−4% (−9; 2)	1% (−1; 2)	−3% (−14; 9)	0% (−5; 6)
**Reproductive hormones**[f]						
Testosterone (nmol/l)	2% (−8; 12)	3% (−2; 8)	−2% (−7; 4)	−2% (−3; 0)	5% (−6; 17)	−4% (−9; 2)
Oestradiol (pmol/l)	18% (−3; 43)	−8% (−17; 1)	10% (−2; 23)	3% (0; 6)	12% (−10; 40)	10% (−2; 23)
SHBG (nmol/l)	−4% (−14; 7)	5% (−1; 12)	−1% (−7; 6)	−5% (−7; −2)	−4% (−15; 9)	−11% (−18; −4)
FSH (IU/l)	−1% (−17; 17)	−1% (−8; 7)	−6% (−15; 4)	2% (−1; 5)	−12% (−28; 7)	7% (−3; 19)
LH (IU/l)	11% (−1; 26)	−7% (−14; 0)	3% (−4; 11)	2% (0; 5)	7% (−7; 23)	4% (−3; 12)
FAI (%)	6% (−4; 18)	−2% (−7; 2)	−1% (−7; 5)	3% (1; 5)	9% (−3; 23)	8% (1; 16)

Results are presented as relative percentage differences. Underweight, overweight, and obese relative to normal weight in participants from the Fetal Programming of Semen Quality (FEPOS) cohort, Denmark, 1998–2019. The direct effect represents the effect of maternal pre-pregnancy BMI not mediated by the sons' birthweight, and the indirect effect represents the effect of maternal pre-pregnancy BMI mediated by the sons' birthweight.
NP, non-progressive motility; IM, immotile; DFI, DNA fragmentation index; HDS, high DNA stainability; SHBG, sex hormone-binding globulin; FAI, free androgen index.

[a] Adjusted for maternal age at delivery, highest parental social class, maternal first-trimester smoking, and alcohol intake.
[b] Further adjusted for abstinence time, spillage, and place of semen sample.
[c] Participants reporting spillage excluded.
[d] Further adjusted for from time from ejaculation to analysis.
[e] Further adjusted for abstinence time.
[f] Further adjusted for time of blood sample.
