# Supplementary material for: Maternal pre-pregnancy BMI and reproductive health in adult sons: a study in the Danish National Birth Cohort
Source: Hum Reprod. 2023 Nov 4;39(1):219–31. doi: 10.1093/humrep/dead230 (PMC10767916; doi:10.1093/humrep/dead230)
Supplement: dead230_Supplementary_Table_S10 [file dead230_supplementary_table_s10.pdf]

OXFORD human reproduction

Human Reproduction, 2023, 1–1
https://doi.org/10.1093/humrep/dead230
Supplementary Data

Supplementary Table S10. Baseline characteristics, potential mediators, and precision variables according to categorizations of paternal BMI in 769 participants from the Fetal Programming of Semen Quality Cohort, Denmark, 1998–2019.

Categorical paternal BMI (pseudo range)	Underweight and normal weight (<25.0)	Overweight (25.0–29.7)	Obese (30.1–39.4)	Missings %
**N (%)**	462 (60.1)	272 (35.4)	35 (4.6)	
**Baseline characteristics**				
**Highest social class of parents**				0
High-grade professional	161 (34.8)	85 (31.3)	7 (20.0)	
Low-grade professional	146 (31.6)	98 (36.0)	14 (40.0)	
Skilled or unskilled worker	128 (27.7)	79 (29.0)	14 (40.0)	
Student or economically inactive	27 (5.8)	10 (3.7)	0 (0.0)	
**Daily number of cigarettes in 1st trimester (maternal)**				0
Non-smoker	370 (80.1)	209 (76.8)	>20[a] (>57.1)	
0–10 cigarettes/day	79 (17.1)	55 (20.2)	10 (28.6)	
>10 cigarettes/day	13 (2.8)	8 (2.9)	<5[a] (<14.3)	
**Alcohol intake in 1st trimester (maternal)**				0
No	225 (48.7)	153 (56.3)	22 (62.9)	
Yes	237 (51.3)	119 (43.8)	13 (37.1)	
**Maternal age at delivery (years), mean (SD)**	31.0 (4.1)	31.1 (4.2)	30.9 (3.8)	0
**Maternal BMI (kg/m^2)**				0
Underweight	31 (6.7)	10 (3.7)	0 (0.0)	
Normal	343 (74.2)	199 (73.2)	>21[a] (>60.0)	
Overweight	70 (15.2)	49 (18.0)	9 (25.7)	
Obese	18 (3.9)	14 (5.1)	<5[a] (<14.3)	
**Potential mediators**				
**Sons' own BMI (kg/m^2)**	21.9 (3.0)	23.0 (3.4)	25.9 (5.4)	<1
**Birth weight (g), mean (SD)**	3718 (530)	3734 (510)	3765 (574)	<2
**Composite marker of pubertal timing, mean (SD)**	2.1 (0.5)	2.0 (0.5)	2.0 (0.4)	<2.5
**Fat mass (kg), mean (SD)**	10.6 (5.9)	12.4 (6.9)	17.7 (11.4)	<1
**Abstinence time in days**				<1
<2	162 (35.1)	89 (33.0)	13 (37.1)	
2–3	147 (31.9)	87 (32.2)	11 (31.4)	
>3	<153[a] (<33.1)	<96[a] (<35.3)	11 (31.4)	
**Spillage**				<1
No	<394[a] (<85.3)	217 (81.3)	29 (82.9)	
Yes	68 (14.8)	50 (18.7)	6 (17.1)	
**Place of semen sample**				<1
At home	59 (12.9)	43 (16.0)	<5[a] (<14.3)	
At clinic	<403[a] (<87.2)	<229[a] (<84.2)	>30[a] (>85.7)	
**Interval between ejaculation and time of analysis**				<1
≤60 min	343 (75.1)	194 (72.7)	29 (82.9)	
>60 min	114 (24.9)	73 (27.3)	6 (17.1)	
**Time of the day of blood sampling**				<1
Morning	176 (38.3)	100 (37.5)	7 (20.0)	
Afternoon	<245[a] (<53.0)	136 (50.9)	19 (54.3)	
Evening	41 (8.9)	31 (11.6)	9 (25.7)	

[a] Local data regulations do not allow reporting of numbers below five. Therefore, some numbers in the table have been masked (</>) to hide numbers smaller than 5.
