# Supplementary material for: Maternal pre-pregnancy BMI and reproductive health in adult sons: a study in the Danish National Birth Cohort
Source: Hum Reprod. 2023 Nov 4;39(1):219–31. doi: 10.1093/humrep/dead230 (PMC10767916; doi:10.1093/humrep/dead230)
Supplement: dead230_Supplementary_Table_S11 [file dead230_supplementary_table_s11.pdf]

Human Reproduction, 2023, 1–1
https://doi.org/10.1093/humrep/dead230
Supplementary Data

Supplementary Table S11. Reproductive health outcomes* according to categorizations of paternal BMI in 769 participants from the Fetal Programming of Semen Quality Cohort, Denmark, 1998–2019.

Categorical maternal pre-pregnancy BMI	Underweight and normal weight (<25.0)	Overweight (25.0–29.7)	Obese (30.1–39.4)	Total
**N (%)**	462 (60.1)	272 (35.4)	35 (4.6)	769 (100.0)
**Semen characteristics**				
Volume (ml)	2.7 (1.8; 3.7)	2.8 (2.1; 3.7)	2.3 (1.9; 3.5)	2.7 (1.9; 3.7)
Concentration (mill/ml)	40.0 (19.7; 75.5)	36.2 (17.0; 73.1)	40.0 (17.2; 62.2)	39.2 (18.8; 74.4)
Total sperm count (mill)	104.1 (52.5; 211.3)	108.0 (41.7; 206.7)	105.2 (28.1; 181.7)	105.1 (46.0; 206.3)
Progressive motility (%)	63.0 (52.0; 74.0)	64.0 (52.4; 74.0)	61.0 (51.5; 68.5)	63.0 (52.0; 74.0)
Morphology (% normal)	6.0 (3.8; 10.0)	5.6 (2.0; 10.0)	9.0 (3.5; 12.7)	6.0 (3.0; 10.0)
DFI (%)	9.0 (6.0; 12.9)	10.0 (7.0; 14.0)	9.8 (5.9; 12.8)	9.0 (7.0; 13.0)
HDS (%)	9.0 (7.0; 12.0)	9.4 (7.0; 14.0)	7.7 (5.7; 10.1)	9.0 (7.0; 13.0)
**Testes volume**				
Average testes volume (ml)	15.0 (12.0; 20.0)	15.0 (11.2; 20.0)	15.2 (12.9; 20.0)	15.0 (12.0; 20.0)
**Reproductive hormones**				
Testosterone (nmol/l)	18.5 (15.0; 22.0)	18.2 (15.1; 22.0)	15.3 (12.2; 22.0)	18.2 (15.0; 22.0)
Oestradiol (pmol/l)	53.2 (34.5; 72.5)	51.9 (34.3; 74.0)	45.9 (29.8; 70.2)	52.3 (34.4; 73.0)
SHBG (nmol/l)	34.0 (26.0; 41.8)	31.2 (24.4; 41.0)	30.3 (21.5; 38.0)	33.0 (25.5; 41.0)
FSH (IU/l)	3.4 (2.4; 5.1)	3.6 (2.5; 5.0)	3.6 (2.3; 5.1)	3.5 (2.4; 5.1)
LH (IU/l)	5.0 (3.9; 6.5)	5.2 (4.3; 6.5)	4.8 (3.6; 6.1)	5.1 (4.0; 6.5)
FAI (%)	55.0 (44.4; 67.0)	57.2 (46.9; 71.9)	55.8 (44.4; 69.6)	55.6 (45.4; 69.5)

* Reproductive health outcomes are presented as pseudo median values (pseudo interquartile range). A pseudo percentile is calculated as the average of the five percentiles nearest the actual percentile to comply with local data regulations.
DFI, DNA fragmentation index; HDS, high DNA stainability; SHBG, sex hormone-binding globulin; FAI, free androgen index.
