# Supplementary material for: Maternal pre-pregnancy BMI and reproductive health in adult sons: a study in the Danish National Birth Cohort
Source: Hum Reprod. 2023 Nov 4;39(1):219–31. doi: 10.1093/humrep/dead230 (PMC10767916; doi:10.1093/humrep/dead230)
Supplement: dead230_Supplementary_Table_S12 [file dead230_supplementary_table_s12.pdf]

OXFORD

human reproduction

Human Reproduction, 2023, 1–1
https://doi.org/10.1093/humrep/dead230
Supplementary Data

Supplementary Table S12. Relative differences in reproductive health outcomes in young adult sons according to categorizations of paternal BMI.

	Overweight		Obese	
	**Crude**	**Adjusted[a] (95% CI)**	**Crude**	**Adjusted[a] (95% CI)**
**Semen characteristics[b]**				
Volume (ml)[c]	2%	1% (−6; 9)	−8%	−2% (−17; 15)
Concentration (mill/ml)	0%	−5% (−17; 9)	−11%	−8% (−30; 22)
Total sperm count (mill)[c]	1%	−5% (−17; 10)	−17%	−12% (−35; 21)
Motility (NP + IM %)[d]	0%	1% (−5; 8)	8%	10% (−2; 24)
Morphology (% normal)	−8%	−11% (−20; 0)	20%	24% (−1; 57)
DFI (%)	7%	4% (−5; 13)	9%	2% (−18; 27)
HDS (%)	5%	4% (−4; 13)	−17%	−19% (−31; −4)
**Testes volume[e]**				
Average testes volume (ml)	−1%	0% (−5; 5)	4%	7% (−3; 19)
**Reproductive hormones[f]**				
Testosterone (nmol/l)	0%	−1% (−5; 4)	−13%	−9% (−20; 4)
Oestradiol (pmol/l)	2%	1% (−9; 11)	−6%	−6% (−25; 16)
SHBG (nmol/l)	−8%	−8% (−17; −2)	−11%	−7% (−18; 6)
FSH (IU/l)	−1%	1% (−8; 10)	1%	3% (−16; 28)
LH (IU/l)	4%	5% (−1; 12)	−6%	−3 (−16; 12)
FAI (%)	9%	8% (2; 14)	−2%	−2% (−13; 11)

Results are presented as relative percentage differences. Overweight and obese relative to normal weight and underweight in 769 participants from the Fetal Programming of Semen Quality (FEPOS) cohort, Denmark, 1998–2019.
NP, non-progressive motility; IM, immotile; DFI, DNA fragmentation index; HDS, high DNA stainability; SHBG, sex hormone-binding globulin; FAI, free androgen index.
[a] Adjusted for maternal age at delivery, highest parental social class, maternal first-trimester smoking, alcohol intake, and maternal pre-pregnancy BMI.
[b] Further adjusted for abstinence time, spillage, and place of semen sample.
[c] Participants reporting spillage excluded.
[d] Further adjusted for from time from ejaculation to analysis.
[e] Further adjusted for abstinence time.
[f] Further adjusted for time of blood sample.
