# Supplementary material for: Maternal pre-pregnancy BMI and reproductive health in adult sons: a study in the Danish National Birth Cohort
Source: Hum Reprod. 2023 Nov 4;39(1):219–31. doi: 10.1093/humrep/dead230 (PMC10767916; doi:10.1093/humrep/dead230)
Supplement: dead230_Supplementary_Table_S13 [file dead230_supplementary_table_s13.pdf]

OXFORD
human reproduction

Human Reproduction, 2023, 1–1
https://doi.org/10.1093/humrep/dead230
Supplementary Data

Supplementary Table S13. Relative differences in reproductive health outcomes in young adult sons according to categorizations of maternal pre-pregnancy BMI.

	Underweight		Overweight		Obese	
	Crude	Adjusted[a] (95% CI)	Crude	Adjusted[a] (95% CI)	Crude	Adjusted[a] (95% CI)
**Semen characteristics**[b]						
Volume (ml)[c]	−13%	−12% (−23; −1)	−11%	−7% (−15; 3)	3%	−5% (−20; 13)
Concentration (mill/ml)	−2%	5% (−17; 34)	−1%	3% (−13; 21)	13%	4% (−20; 37)
Total sperm count (mill)[c]	−3%	−9% (−31; 20)	−14%	−11% (−24; 5)	24%	11% (−16; 47)
Motility (NP + IM %)[d]	3%	2% (−12; 18)	−1%	−2% (−9; 6)	9%	10% (−3; 24)
Morphology (% normal)	10%	12% (−6; 34)	4%	8% (−6; 24)	13%	14% (−8; 41)
DFI (%)	−13%	18% (−4; 45)	−5%	−5 (−15; 6)	2%	−4% (−25; 23)
HDS (%)	1%	−3% (−18; 16)	3%	−1% (−10; 9)	−4%	−4% (−20; 16)
**Testes volume**[e]						
Average testes volume (ml)	−4%	−8% (−18; 4)	−3%	−5% (−11; 1)	−4%	0% (−13; 15)
**Reproductive hormones**[f]						
Testosterone (nmol/l)	5%	6% (−4; 17)	−5%	−2% (−8; 4)	2%	4% (−6; 14)
Oestradiol (pmol/l)	8%	18% (−5; 46)	9%	9% (−4; 25)	13%	30% (10; 53)
SHBG (nmol/l)	1%	−3% (−21; 20)	−6%	−1% (−8; 6)	−14%	−11% (−24; 4)
FSH (IU/l)	−7%	−8% (−25; 12)	−2%	−4% (−14; 9)	−9%	−5% (−20; 12)
LH (IU/l)	2%	−1% (−10; 10)	5%	3% (−5; 11)	8%	10% (−1; 23)
FAI (%)	15%	9% (−9; 31)	0%	−1% (−7; 6)	18%	17% (5; 31)

Results are presented as relative percentage differences. Underweight, overweight, and obese relative to normal weight in 769 participants from the Fetal Programming of Semen Quality (FEPOS) cohort, Denmark, 1998–2019, with further adjustment for paternal BMI.
NP, non-progressive motility; IM, immotile; DFI, DNA fragmentation index; HDS, high DNA stainability; SHBG, sex hormone-binding globulin; FAI, free androgen index.

[a] Adjusted for maternal age at delivery, highest parental social class, maternal first-trimester smoking, alcohol intake, and paternal BMI at 18 months.
[b] Further adjusted for abstinence time, spillage, and place of semen sample.
[c] Participants reporting spillage excluded.
[d] Further adjusted for from time from ejaculation to analysis.
[e] Further adjusted for abstinence time.
[f] Further adjusted for time of blood sample.
